# Thiolated Chitosan Masked Polymeric Microspheres with Incorporated Mesocellular Silica Foam (MCF) for Intranasal Delivery of Paliperidone

**DOI:** 10.3390/polym9110617

**Published:** 2017-11-15

**Authors:** Stavroula Nanaki, Maria Tseklima, Evi Christodoulou, Konstantinos Triantafyllidis, Margaritis Kostoglou, Dimitrios N. Bikiaris

**Affiliations:** 1 Laboratory of Polymer Chemistry and Technology, Aristotle University of Thessaloniki, GR-54124 Thessaloniki, Greece; sgnanaki@chem.auth.gr (S.N.); mtseklima@pharmathen.com (M.T.); evicius@gmail.com (E.C.); 2 Laboratory of General and Inorganic Chemical Technology, Aristotle University of Thessaloniki, GR-54124 Thessaloniki, Greece; ktrianta@chem.auth.gr (K.T.); kostoglu@chem.auth.gr (M.K.)

**Keywords:** PLA, PLGA, mesoporous cellular foam, paliperidone, thiolated chitosan, microspheres, drug encapsulation

## Abstract

In this study, mesocellular silica foam (MCF) was used to encapsulate paliperidone, an antipsychotic drug used in patients suffering from bipolar disorder. MCF with the drug adsorbed was further encapsulated into poly(lactic acid) (PLA) and poly(lactide-*co*-glycolide) (PLGA) 75/25 *w*/*w* microspheres and these have been coated with thiolated chitosan. As found by TEM analysis, thiolated chitosan formed a thin layer on the polymeric microspheres’ surface and was used in order to enhance their mucoadhesiveness. These microspheres aimed at the intranasal delivery of paliperidone. The DSC and XRD studies showed that paliperidone was encapsulated in amorphous form inside the MCF silica and for this reason its dissolution profile was enhanced compared to the neat drug. In coated microspheres, thiolated chitosan reduced the initial burst effect of the paliperidone dissolution profile and in all cases sustained release formulations have been prepared. The release mechanism was also theoretically studied and three kinetic models were proposed and successfully fitted for a dissolution profile of prepared formulations to be found.

## 1. Introduction

Mesoporous silica nanoparticles (MSN) are among a variety of drug delivery systems that have been tested extensively in recent years for the delivery of water-insoluble drugs [1]. Due to their large surface areas and porous interiors they can be used as reservoirs to store hydrophobic drugs. The pore size and environment can be tailored to selectively store different molecules of interest [2,3], while the size and shape of the particles can be tuned to maximize cellular uptake. Such silica-based materials have been successfully used as drug-delivery vectors [4,5], gene transfection reagents [6], cell markers [7], and carriers of molecules [8]. Mesocellular foam (MCF) silica particles have an almost spherical morphology and a continuous three-dimensional pore system, with pore size in the range of ca. 10–30 nm, having already been used for delivery of drugs [9,10] through oral [11,12,13] and intravenous [14,15] routes.

Paliperidone is a second-generation antipsychotic drug and is effective in treating both positive and negative symptoms of schizophrenia with an increased safety effect towards extrapyramidal symptoms. For that, paliperidone is administrated in two forms: in oral form, present on the market as a 24-h extended-release tablet [16], and in injectable form [17], marketed by Janssen (Invega Sustenna, Titusville, NJ, USA). Such long active injectables (LAI) have gained attention recently for their promise to treat diseases like schizophrenia. However, paliperidone is a poorly water soluble drug, which limits its effectiveness. For this reason, in our previous study [18] it was first adsorbed into MCF silica and then encapsulated in polymeric microparticles. Poly(l-lactic acid) (PLA) [19,20] and poly(d,l-lactide-*co*-glycolide) (PLGA) [21] are two Food and Drug Administration (FDA)-approved polymers that are used particularly in nasal application of active compounds. These polymers are transformed into lactic and/or glycolic acid in the body due to hydrolysis. However, when Darville et al. [17] investigated the local disposition of paliperidone in rats, they found that drug release caused a reaction leading to chronic inflammation. Also, large amounts of crystalline paliperidone-LAI particles were found within the infiltrating macrophages, supporting the hypothesis that the sudden drop in dissolution rate of the drug and its potential absorption lead to accumulation of the drug in macrophages. The same group investigated the co-administration of liposomal clodronate and sunitinib in order to inhibit the depot infiltration and nano-/microparticle phagocytosis by macrophages, and the neovascularization of the depot, respectively [22].

In recent years, the nasal route has gained importance as a non-invasive drug application route that offers many advantages for the introduction of drugs into systemic circulation, such as rapid absorption of drugs and therefore quick onset of their effect, and avoidance of the hepatic first-pass effect. However, disadvantages of the nasal route include enzymatic barriers, particularly in the case of macromolecular drugs, and the low permeability of the nasal epithelia. Furthermore, it is necessary that any formulation intended for nasal delivery must have strong mucoadhessive properties. Chitosan is a positively charged polymer [23] frequently used in nasal application of macromolecules [24,25], due to its strong mucoadhesive properties and its ability to transiently open the tight junctions in the nasal mucosa. Mucoadhesion is achieved by the ionic interaction of positively charged amine groups of d-glucosamine units of chitosan with negatively charged sialic acid groups of musin or other negatively charged groups of the mucosal membrane [26]. It has been reported that chitosan does not lead to any histological changes in the nasal mucosa [27,28,29]. So in order to increase the mucoadhesive properties of PLA or PLGA microspheres, their surface could be coated with chitosan. Moreover, from the literature it was found that thiolation of chitosan shows significant improvement in permeation and better mucoadhesive properties than neat chitosan due to the fact that the covalent bonds formed between the thiol group and the mucus glycoprotein are stronger than the noncovalent bonds between chitosan and mucus glycoprotein [30,31,32,33,34,35].

The aim of the present study was to enhance the solubility of paliperidone by its adsorption in MCF particles and to prepare proper microspheres for intranasal delivery of the drug. Microparticles prepared expected to act as extended release formulations of the drug, lasting for about one month. In order to be administrated by the intranasal route, thiolated chitosan was used as a coating membrane. Thiolated chitosan, apart from mucoadhesion enhancement, has the additional advantage of increasing drug penetration into the nasal mucous membrane. As far as we know, no other study has been published concerning the intranasal delivery of paliperidone via thiolated masked polymeric microparticles.

## 2. Materials and Methods 

### 2.1. Materials and Reagents

Pluronic P-123 (Poly(ethylene glycol)-block-poly(propylene glycol)-block-poly(ethylene glycol)) triblock copolymer with average *M*_n_~5800 was acquired from Sigma-Aldrich (St. Louis, MO, USA) and was used as the MCF mesostructure-directing agent, together with 1,2,3-trimethylbenzene (TMB, Fluka, Munich, Germany), which was utilized as a co-surfactant and swelling agent, as well as ammonium fluoride (NH_4_F, Merck, Kenilworth, NJ, USA) serving as a mineralizing agent [36]. Tetraethyl orthosilicate (TEOS) was acquired from Merck and used as the silica source of MCF. Chitosan low molecular weight (50,000–190,000 Da, degree of deacetylation ≥ 75%) was purchased from Sigma-Aldrich. Thioglycolic acid (purum ≥ 98%), *N*-Ethyl-*N*′-(3-dimethylaminopropyl) carbodiimide hydrochloride (EDAC·HCl) (purum ≥ 99%) and *N*-Hydroxysuccinimide (NHS) (purum 98%) were purchased from Sigma-Aldrich (St. Louis, MO, USA). Poly(lactic acid) (PURASORB PDL 02 with an inherent viscosity midpoint of 0.2 dL/g), and Poly(lactide-*co*-glycolide) 75/25 *w*/*w* copolymer (PURASORB PDLG 7502 with an inherent viscosity midpoint of 0.2 dL/g) were kindly donated by Corbion (Montmelo, Spain). Paliperidone was kindly donated by Pharmathen S.A (Athens, Greece). All other reagents were of analytical grade.

### 2.2. Synthesis of MCF 

MCF was previously synthesized in our lab using the self-assembly method described by Winkel et al. [18,36]. In brief, Pluronic P-123 was used as the structure directing agent, 1,2,3-trimethylbenzene (TMB) was used as the swelling agent, ammonium fluoride (NH_4_F) as the mineralizing agent, and tetraethyl orthosilicate (TEOS) as the silica source, in acidic pH conditions. P-123 was dissolved in aqueous HCl 1.6 M followed by the addition of NH_4_F and TMB, and the mixture was stirred for 1 h at 40 °C. TEOS was then added to the solution and stirring continued for 20 h at 40 °C. The resulting mixture was transferred into an autoclave and heated at 100 °C for 24 h. Filtration was used to recover the solid products, followed by a washing step with water, and calcination was conducted in air at 500 °C, for 8 h, with a heating rate of 1 °C/min, in order to combust the organic templates.

### 2.3. Synthesis of Thiolated Chitosan

Thiolated chitosan (TMC) was synthesized by a two-stage procedure reported by Zhu et al. [37]. In brief, 1 mL thioglycolic acid, 3.5 mg EDAC·HCl, and 2.0 mg NHS were inserted into a flask containing 2 mL DMF and the mixture was left under magnetic stirring overnight, resulting in the synthesis of NHS–ester as an intermediate product. In the second stage, 500 mg of low molecular weight chitosan was added to 4 mL hydrochloric solution, 1 M in concentration, and diluted with water to a final concentration of 2.5%. After that, NHS–ester was inserted dropwise into the chitosan solution, and the pH value was adjusted to 5. The resulting mixture was left under magnetic stirring overnight. Synthesized TMC was lyophilized and washed in a Soxhlet extractor until total elimination of unreacted monomers was achieved. After lyophilization, TMS appeared as a white, odorless solid with a fibrous structure. Its successful synthesis was evaluated by FTIR.

### 2.4. Paliperidone Loading Procedure on MCF

Paliperidone was loaded on MCF by adsorption [18]. In brief, the proper quantity of the drug was dissolved in a mixture of organic solvents isopropanol/dichloromethane 75/25 *v*/*v*, resulting in a solution of 0.1% *w*/*v*. Mesoporous silica MCF (100 mg) was added to the organic solution and the resulting dispersion was left under magnetic stirring for 24 h, in a nitrogen atmosphere. MCF with paliperidone adsorbed was isolated by centrifugation at 4000 rpm for 10 min and at room temperature until total evaporation of the solvent was achieved. The dried material was washed with acetone to remove the quantity of paliperidone that was deposited onto MCF’s surface. The amount of the adsorbed drug was determined by TGA and BET.

### 2.5. Preparation of PLA and PLGA Microspheres Loaded with MCF/Paliperidone

Polymeric microspheres (PLA and PLGA) containing MCF with adsorbed paliperidone were prepared by the solid-oil-water (s/o/w) modified double emulsification method [38]. According to this procedure, 100 mg of polymer, PLA or PLGA 75/25 *w*/*w* were dissolved in 5 mL of dichloromethane. Ten milligrams of MCF containing adsorbed paliperidone were added to the polymeric solution and dispersed using a probe sonicator for 1 min. The dispersion was inserted dropwise in 100 mL of PVA solution, 1% *w*/*v* in concentration, and homogenized using a homogenizer for 2 min. After that, 100 mL water were added and the mixture was left under magnetic stirring until the total evaporation of dichloromethane was achieved. After centrifugation at 8000 rpm for 10 min, the microspheres were collected and washed three times with distilled water in order to remove traces of the residual solvent and PVA. The resulting microspheres were finally freeze-dried and stored at 4 °C for further evaluation.

### 2.6. Preparation of Thiolated Coated PLA and PLGA Microspheres

Microspheres prepared via the above stages were further modified with thiolated chitosan according to the procedure described by Jiang et al. [39]. In brief, pre-weighted thiolated chitosan was dissolved in deionized water at a concentration of 0.025 mg/mL. The prepared PLA and PLGA 75/25 *w*/*w*/microspheres loaded with MCF/paliperidone were suspended in a thiolated chitosan solution at a concentration of 9.5 mg/mL by sonication for 1 min at 30 W power output. They were left under magnetic stirring for 15 min and then collected by centrifugation at 80,000× *g* for 15 min. The modified microspheres were washed with water once and then collected by freeze drying. They were stored in a vacuum until further use.

MCF with loaded paliperidone was also masked with thiolated chitosan for comparison reasons following the procedure described above. All the formulations were fully characterized.

### 2.7. Characterization of Prepared Formulations

#### 2.7.1. Morphology, Thermal Properties and Crystallinity of Prepared Formulations

Fourier Transform-Infrared spectroscopy (FTIR) spectra were obtained on a Perkin-Elmer FTIR spectrometer (Spectrum 1, Waltham, MA, USA) using pellets of MCF and MCF-Paliperidone diluted in KBr. Infrared (IR) absorbance spectra were obtained between 450 and 4000 cm^−1^ at a resolution of 4 cm^−1^ using 20 co-added scans. All spectra presented are baseline-corrected and normalized.

X-ray diffraction (XRD) analysis was performed on MCF silica and MCF-paliperidone over the 5–45° 2θ range, using a MiniFlex II diffractometer (Rigaku Co., Tokyo, Japan) with Bragg–Brentano geometry (θ, 2θ) and Ni-filtered Cu Kα radiation (λ = 0.154 nm).

Thermogravimetric analysis (TGA) was carried out with a SETARAM SETSYS TG-DTA 16/18 (Caluire, France). Samples (6.0 ± 0.2 mg) were placed in alumina crucibles. An empty alumina crucible was used as a reference. Paliperidone, MCF, and MCF-Paliperidone were heated from ambient temperature to 600 °C in a 50 mL/min flow of N_2_ at heating rate of 20 °C/min.

A Perkin-Elmer Pyris 1 differential scanning calorimeter (DSC) (Waltham, MA, USA), calibrated with Indium and Zinc standards was employed. A sample of about 10 mg was used for each test, placed in scaled aluminum pan and heated to 300 °C at a heating rate of 20 °C/min. The sample was held at that temperature for 5 min in order to erase any thermal history. After that it was quenched to 30 °C with liquid nitrogen and scanned immediately to 300 °C at a heating rate of 20 °C/min.

The morphology of the prepared microspheres was examined using a scanning electron microscope (SEM), type Jeol (JMS-840, Peabody, MA, USA). All the studied surfaces were coated with carbon black to avoid charging under the electron beam. SEM photos were also received after a dissolution study.

Transmittance electron microscopy (TEM) experiments were carried out on a JEOL 2011 TEM (Peabody, MA, USA) with a LaB6 filament and an accelerating voltage of 200 kV. The specimens were prepared by evaporating drops of SBA-15 silica–ethanol suspension after sonication onto a carbon-coated lacy film supported on a 3 mm diameter, 300 mesh copper grid.

#### 2.7.2. High-Pressure Liquid Chromatography (HPLC) Quantitative Analysis and Drug Loading

Quantitative analysis and drug loading was performed using a Shimadzu HPLC (Kyoto, Japan) prominence system consisting of a degasser (DGU-20A5), a liquid chromatograph (LC-20 AD), an auto sampler (SIL-20AC), a UV/Vis detector (SPD-20A) and a column oven (CTO-20AC). For the analysis a validated method was used [40]. In detail, a C18 reversed-phase column (250 mm × 4.6 mm i.d., 5-μm particle) was used; the mobile phase was water (pH = 3.5):methanol 70:30 *v*/*v* and the flow rate was 1 mL·min^−1^. UV detection was performed at 275 nm. In brief, the proper amount of microspheres was dissolved in dichloromethane and left under magnetic stirring overnight. The samples were filtered through a 0.45-μm membrane and quantified by HPLC.

Microparticle yield (%), drug loading (%), and entrapment efficiency (%) were calculated by the following equations:

(1) $$\mathrm{Microparticles}\;\mathrm{yield}\;(\%)=\frac{\mathrm{weight}\;\mathrm{of}\;\mathrm{microparticles}}{\mathrm{weight}\;\mathrm{of}\;\mathrm{polymer}\;\mathrm{and}\;\mathrm{drug}\;\mathrm{fed}\;\mathrm{initially}}\cdot 100$$

(2) $$\mathrm{Drug}\;\mathrm{loading}\;\mathrm{content}\;(\%)=\frac{\mathrm{weight}\;\mathrm{of}\;\mathrm{drug}\;\mathrm{in}\;\mathrm{microparticles}}{\mathrm{weight}\;\mathrm{of}\;\mathrm{microparticles}}\cdot 100$$

(3) $$\mathrm{Entrapment}\;\mathrm{efficiency}\;(\%)=\frac{\mathrm{weight}\;\mathrm{of}\;\mathrm{drug}\;\mathrm{in}\;\mathrm{microparticles}}{\mathrm{weight}\;\mathrm{of}\;\mathrm{drug}\;\mathrm{fed}\;\mathrm{initially}}\cdot 100$$

#### 2.7.3. In Vitro Release Profile

In vitro release rates of paliperidone from the prepared formulations were measured in USP dissolution apparatus I (basket apparatus). The dissolution apparatus used was a DISKTEK 2100 C (Markham, ON, Canada) with an auto sampler DISTEK EVOLUTION 4300 and a DISKTEK syringe pump. Dissolution tests were performed in 900 mL simulated body fluid used as dissolution medium having pH 7.2 and temperature 37 ± 1 °C. The rotation speed was set at 50 rpm.

## 3. Results and Discussion

### 3.1. Characterization of MCF/Paliperidone-Loaded Nanoparticles

MCF silica was previously synthesized in our lab and porosimetry studies showed that the N_2_ adsorption isotherms of the parent MCF were of type IV according to the IUPAC classification [18], being characteristic of this type of mesoporous materials. The BET method showed that MCF has a relative high specific surface area, 445 m^2^/g, compared to classical synthesized silica particles, and its average mesopore size (diameter) was about 15 nm, a pore size ideal for paliperidone adsorption. TEM images (shown in our previous work) verified its cellular pore morphology.

MCF was also characterized after the adsorption of paliperidone [18]. In brief, the BET method showed that MCF, after paliperidone’s adsorption, showed a reduced specific surface area of 232 m^2^/g, with its total pore volume being reduced from 1.216 to 0.873 cc/g, showing that paliperidone had been successfully inserted into its pores. Furthermore, thermogravimetric analysis (TGA) was used to determine the drug content loaded on the MCF particles, which was found to be 23.78 wt % [18].

### 3.2. Characterization of Thiolated Chitosan

**Figure 1:**
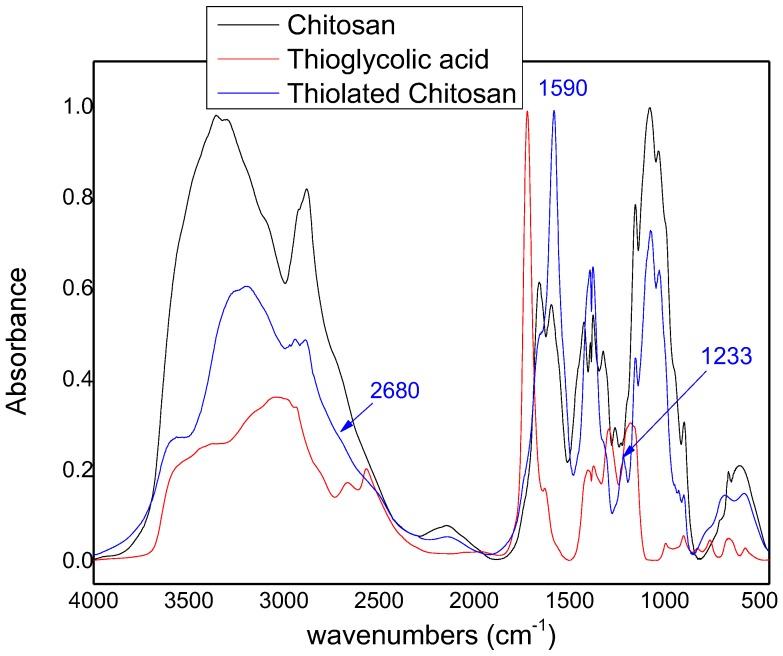
FTIR spectra of chitosan, thioglycolic acid, and thiolated chitosan.

Synthesis of thiolated chitosan was evaluated using FTIR. As can be seen in Figure 1, thiolated chitosan showed some characteristic peaks from the newly formed amide bond at 1590 cm^−1^ and peaks of thiol groups; one at 1233 cm^−1^ owning to the S–C bond and one small peak (which presents as a shoulder) in 2680 cm^−1^ owing to the H–S bond [37]. The existence of these peaks showed the successful synthesis of thiolated chitosan.

**Figure 2:**
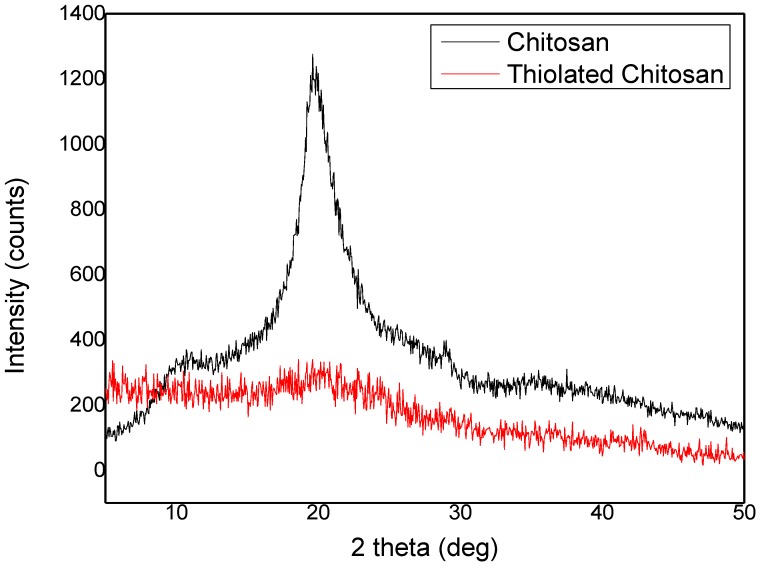
XRD patterns of chitosan and thiolated chitosan.

These changes in the chemical form of chitosan affected its physical stage. XRD patterns (Figure 2) showed that chitosan has a wide angle with high intensity at 19.78°, attributed to its crystalline nature. This peak disappeared in thiolated chitosan, probably due to the decrease in the amount of the free amino groups after the introduction of thiol groups, which significantly reduce intermolecular hydrogen bonds. This reduces the ability of chitosan to be crystallized. The absence of a specific peak also illustrates that thiolated chitosan was successfully synthesized and is completely amorphous, as was already reported in the literature [41].

**Figure 3:**
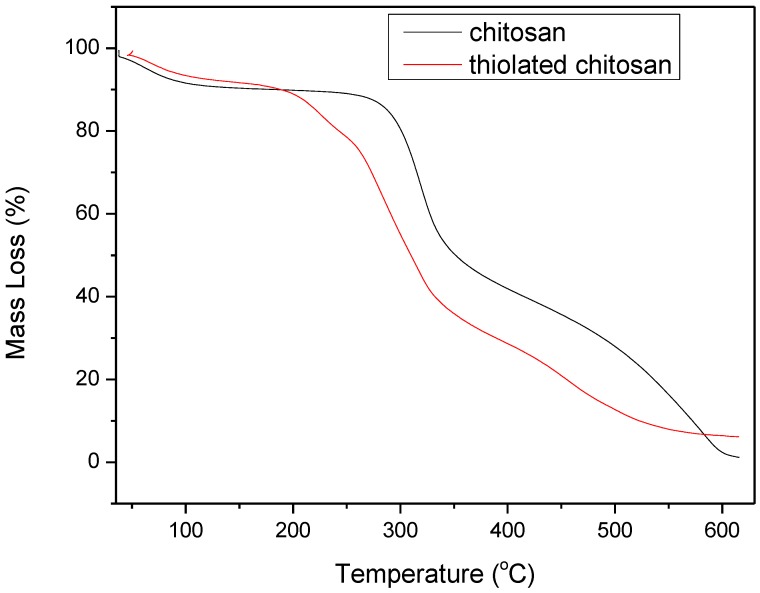
TGA thermograms of chitosan and thiolated chitosan.

A TGA thermogram of chitosan and thiolated chitosan (Figure 3) showed a first stage weight loss up to 105 °C, attributed to the water adsorbed or bound to the polymers, showing weight losses of 10% and 8%, respectively. No mass loss was observed up to 255 °C for chitosan, while after that, a second stage weight loss of 45% was recorded up to 350 °C. This stage is probably due to the dehydration of the saccharide rings, depolymerization, and decomposition of the acetylated and deacetylated units of the chitosan [42,43]. A third stage was recorded up to 600 °C, at which chitosan was completely decomposed. Thiolated chitosan showed a second stage that ranged between 105 and 255 °C, with a weight loss equal to 15% probably due to the degradation of thiol groups in thiolated chitosan. From that temperature to 350 °C, a third stage of thermal decomposition, similar to the second stage decomposition of chitosan, was recorded, corresponding to 40% mass loss and attributed to the dehydration of the saccharide rings, depolymerization, and decomposition of the acetylated and deacetylated units of the chitosan. A final stage up to 600 °C was recorded and led to complete decomposition of thiolated chitosan.

### 3.3. Characterization of MCF-Paliperidone Nanoparticles Coated with Thiolated Chitosan

**Figure 4:**
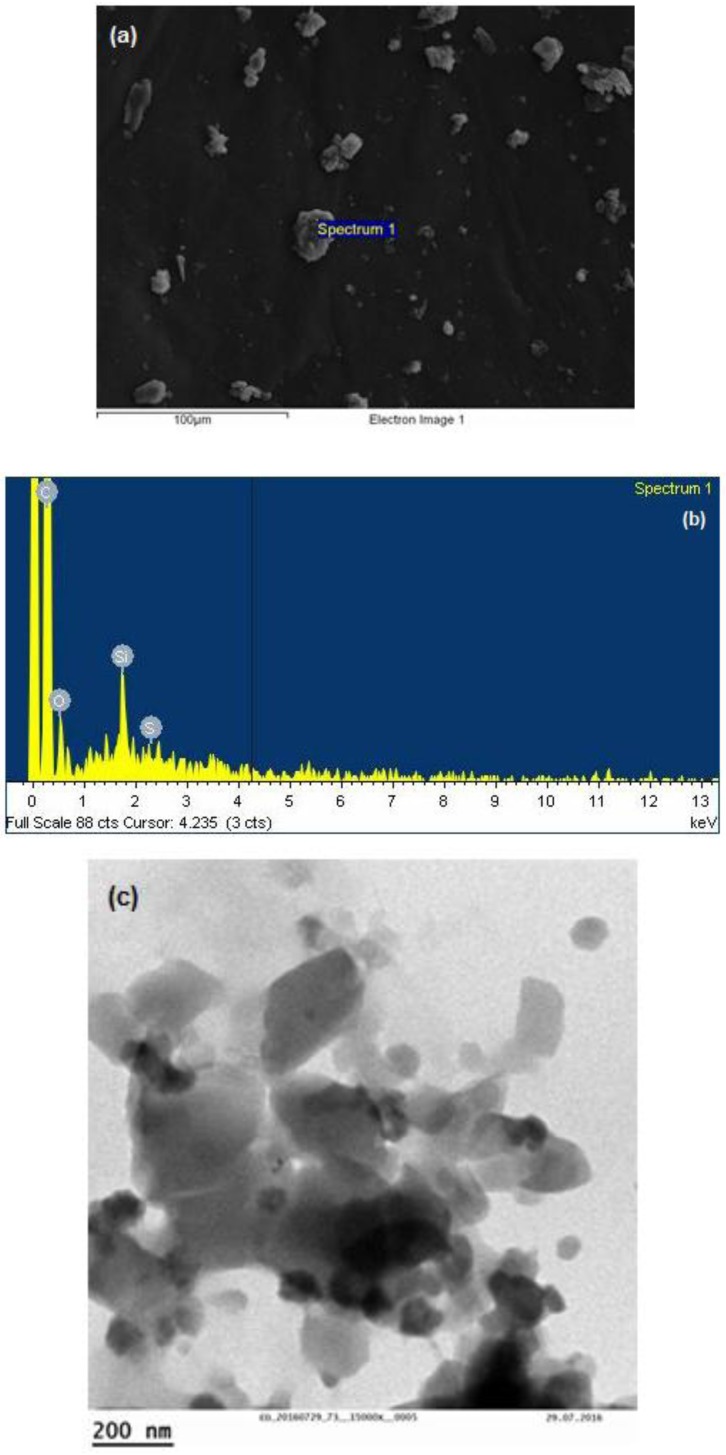
MCF/paliperidone-loaded nanoparticles coated with thiolated chitosan: (**a**) SEM micrograph, (**b**) EDX analysis, and (**c**) TEM micrograph.

The synthesized thiolated chitosan was first used as a coating polymer for MCF/paliperidone-loaded particles. SEM and TEM micrographs were taken in order to examine their morphology. As can be seen in Figure 4a, the primary nanoparticles are aggregated in microparticles with irregular shapes and from EDX analysis (Figure 4b) it is detected that they have both Si and S elements, confirming the presence of MCF and thiolated chitosan, respectively. From the TEM images (Figure 4c) it is evident that the morphology of the primary MCF silica nanoparticles was similar to that of the parent MCF and thiolated chitosan covered these nanoparticles [18].

**Figure 5:**
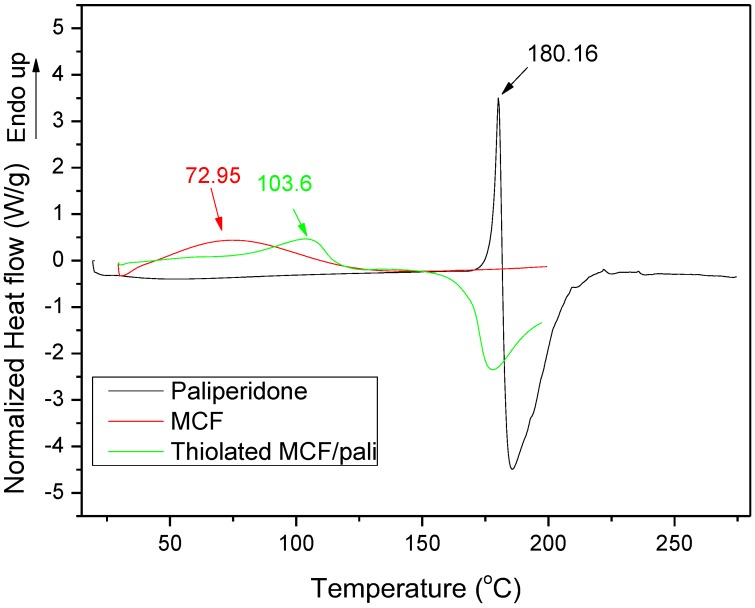
DSC thermograms of paliperidone, MCF, and thiolated MCF with adsorbed paliperidone.

As was found from XRD studies, the incorporation of paliperidone into MCF pores leads to drug amorphization [18]. This also happened when the loaded particles were coated with thiolated chitosan and in XRD patterns no characteristic crystalline peaks were observed (data not shown) [18]. This amorphization was also verified by DSC. As can be seen from Figure 5, neat paliperidone has a melting point of 180.16 °C. After its encapsulation into MCF particles and coating with thiolated chitosan, this peak has disappeared, showing that paliperidone is in a totally amorphous state. A small peak was recorded at 103.6 °C, maybe due to the water removal, which was also recorded in neat MCF particles but at a much lower temperature (72.95 °C). The small difference between the two materials could be due to the existence of thiolated chitosan in the coated MCF, which is also a hydroscopic material and absorbs more water than neat MCF.

### 3.4. Characterization of Polymeric PLA and PLGA Microspheres before and after Coating with Thiolated Chitosan

**Figure 6:**
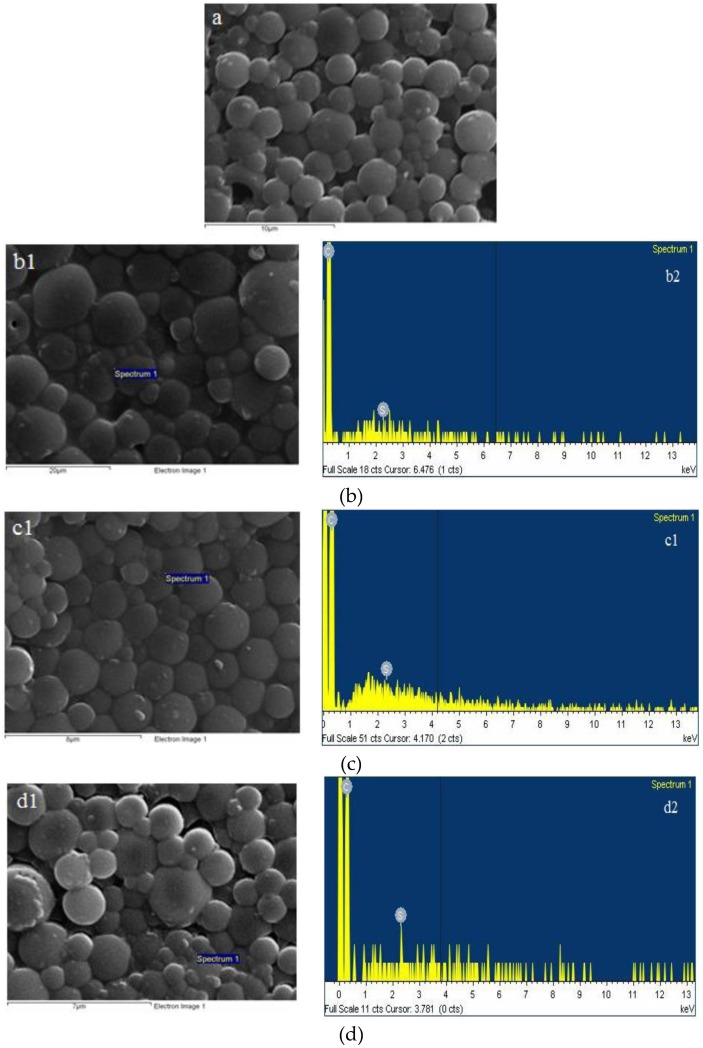
SEM images of prepared microspheres: (**a**) Thiolated_PLA_Pal, (**b**) Thiolated_PLA_MCF_Pal, (**c**) Thiolated_PLGA75/25_Pal, and (**d**) Thiolated_PLGA75/25_MCF_Pal.

The incorporation of paliperidone into MCF nanoparticles was done in order to prepare a completely amorphous drug and thus increase its dissolution profile [18]. It is well known that amorphous drugs have in some cases 1000 times higher solubility compared with their crystalline forms [44,45]. However, these systems lead to immediate release formulations, which are inappropriate for paliperidone drug release. For this reason, MCF/paliperidone-loaded nanoparticles have been microencapsulated into PLA and PLGA microspheres and given controlled release formulations [18]. In the present work we have extended our previous study by preparing the same microspheres but coated with thiolated chitosan, in order to increase the mucoadhesive properties of their surfaces. Also, the previous microspheres were appropriate for injectable formulations and have recently been prepared for intranasal delivery. The successful coating, as well as the morphology of neat and modified microparticles with thiolated chitosan, was verified with SEM and TEM techniques. As can be seen in Figure 6, in all cases microspheres with spherical shapes formed. Thiolated/PLA/Pal microspheres have sizes varying between 1 and 3 μm, while after MCF incorporation the proper microparticles, i.e., Thiolated_PLA_MCF_Pal, showed bigger sizes that varied between 6 and 10 μm. Analogous observations were made for microspheres prepared with PLGA copolymer, since Thiolated_PLGA75/25_Pal and Thiolated_PLGA75/25_MCF_Pal have sizes between 2–4 μm and 3–6 μm, respectively. This observation is probably due to MCF incorporation, which leads to microspheres with bigger sizes. Another observation is that microspheres prepared with PLGA have smaller sizes than those prepared with PLA, probably due to glycolic acid entanglements resulting in the formation of tighter segments.

**Figure 7:**
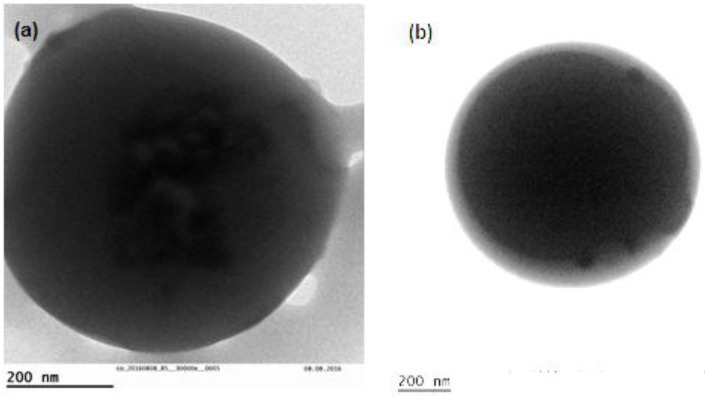
TEM images of microspheres: (**a**) Thiolated_PLA_MCF_Pal and (**b**) Thiolated_PLGA75/25_MCF_Pal.

TEM images verified the successful incorporation of MCF/paliperidone nanoparticles inside of formed microspheres. As can be seen from Figure 7a, MCF with adsorbed paliperidone were detected inside the microspheres, while some of them were also located on the microsphere’s surface (Figure 7b). Also, it is clear that thiolated chitosan forms a thin coating layer on the microsphere surface, which ranged between 20 and 50 nm (Figure 7b).

**Figure 8:**
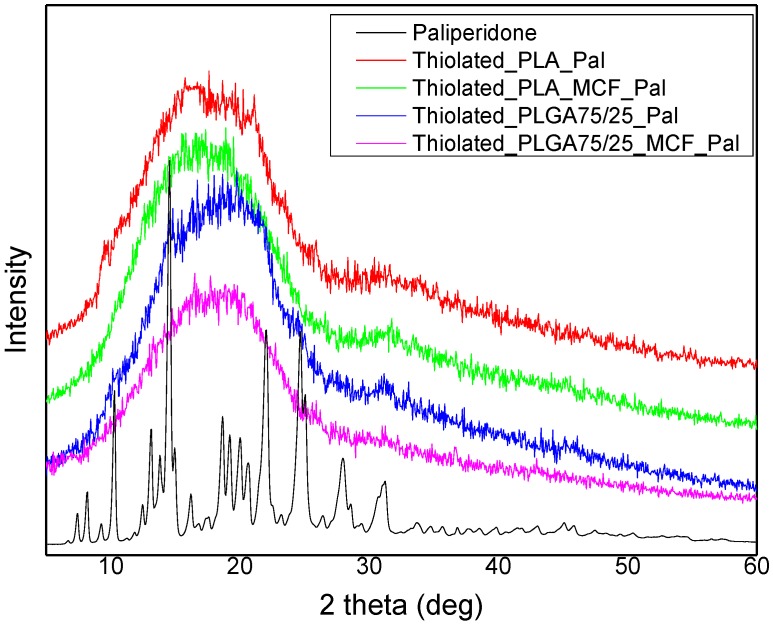
XRD patterns of thiolated microspheres loaded with paliperidone.

The physical state of encapsulated MCF/paliperidone nanoparticles in PLA and PLGA microspheres was studied with XRD. From the recorded patterns it can be seen that all microparticles are amorphous, since only a very broad peak was recorded (Figure 8). This was expected since both PLA and PLGA form amorphous microspheres, while paliperidone was also incorporated in an amorphous form inside the MCF pores [18].

**Figure 9:**
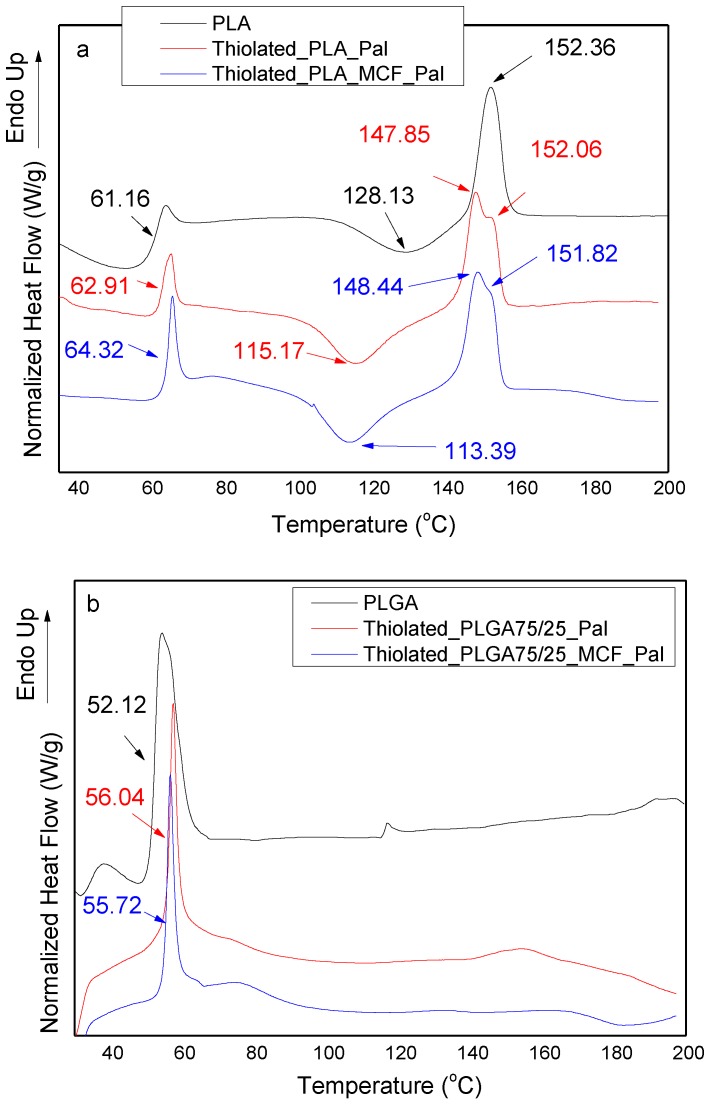
DSC thermograms of thiolated chitosan coated microspheres consisted from (**a**) PLA and (**b**) PLGA with encapsulated paliperidone and MCF_paliperidone.

DSC studies were also conducted in order to examine the crystallinity and structure behavior of prepared PLA and PLGA microspheres loaded with paliperidone and MCF/paliperidone and coated with thiolated chitosan. Figure 9a shows a DSC thermogram of neat PLA and thiolated microspheres of PLA_Pal and PLA_MCF_Pal. As can be seen, PLA exhibits a glass transition temperature of 61.16 °C. Microspheres masked with thiolated chitosan showed some changes concerning the recorded *T*_g_ value, indicating that the addition of both paliperidone and MCF loaded with paliperidone can affect the mobility of PLA chains. For this reason, the *T*_g_ value in Thiolated_PLA_Pal changed to 62.91 °C, while this value shifted to 64.32 °C in Thiolated_PLA_MCF_Pal. However, these chances must be attributed to the incorporation of inorganic nanoparticles into a polymer matrix rather than the addition of a thiolated layer on the microparticle surface. So it is clear that the addition of inorganic nanoparticles causes a higher reduction of chain mobility, which is in agreement with the literature [18]. At higher temperatures PLA can be crystallized; a cold crystallization (*T*_cc_) peak was recorded at 128.13 °C and formed crystals are melted (*T*_m_) at 152.36 °C. These temperatures were also affected by MCF/drug incorporation. *T*_cc_ was shifted to 115.17 °C and in Thiolated_PLA_Pal and to 113.39 °C in Thiolated_PLA_MCF_Pal microspheres. This can be attributed mainly to the crystallization effect of both the drug and MCF on PLA crystallization, which can act as nucleating agents [46] and not to coating layer of thiolated chitosan. A small effect was also found on the melting temperature of PLA: two peaks are present in thiolated microspheres loaded with the drug and MCF/paliperidone, probably due to the formation of two types of crystals. In addition, no further peak owing to MCF and/or paliperidone was present in the DSC thermogram, indicating that MCF with paliperidone adsorbed is encapsulated in an amorphous state in microspheres. This observation is in accordance with the results found by XRD analysis.

Some changes can also be seen in the DSC thermograms of thiolated microspheres prepared with PLGA 75/25 *w*/*w* copolymer (Figure 9b). As can be seen, PLGA is amorphous, showing a *T*_g_ value at 52.12 °C, while coated microspheres with encapsulated paliperidone and MCF/paliperidone showed a slight increase in the *T*_g_ value up to about 56 °C, indicating a slight decrease in the mobility of PLGA chains. A similar shift was also recorded in PLA microspheres, as discussed previously. However, as can be seen, PLGA cannot be crystallized and remains in an amorphous form without recording any *T*_cc_ and *T*_m_ temperatures, as in the case of PLA.

### 3.5. Dissolution Study of Prepared Microspheres

Table 1 shows the drug loading, entrapment efficiency, and microparticle yield of all prepared formulations. As can be seen, all prepared microspheres showed particularly high yield values higher than 86%. Microparticles containing MCF appeared to have higher values than ones prepared with paliperidone alone, maybe due to the incorporation of MCF inside the microparticles. Drug loading values showed a similar behavior, and those with MCF have higher values compared to microparticles with an encapsulated neat drug. Finally, the entrapment efficiency was higher in copolymers, compared to PLA microparticles.

**Figure 10:**
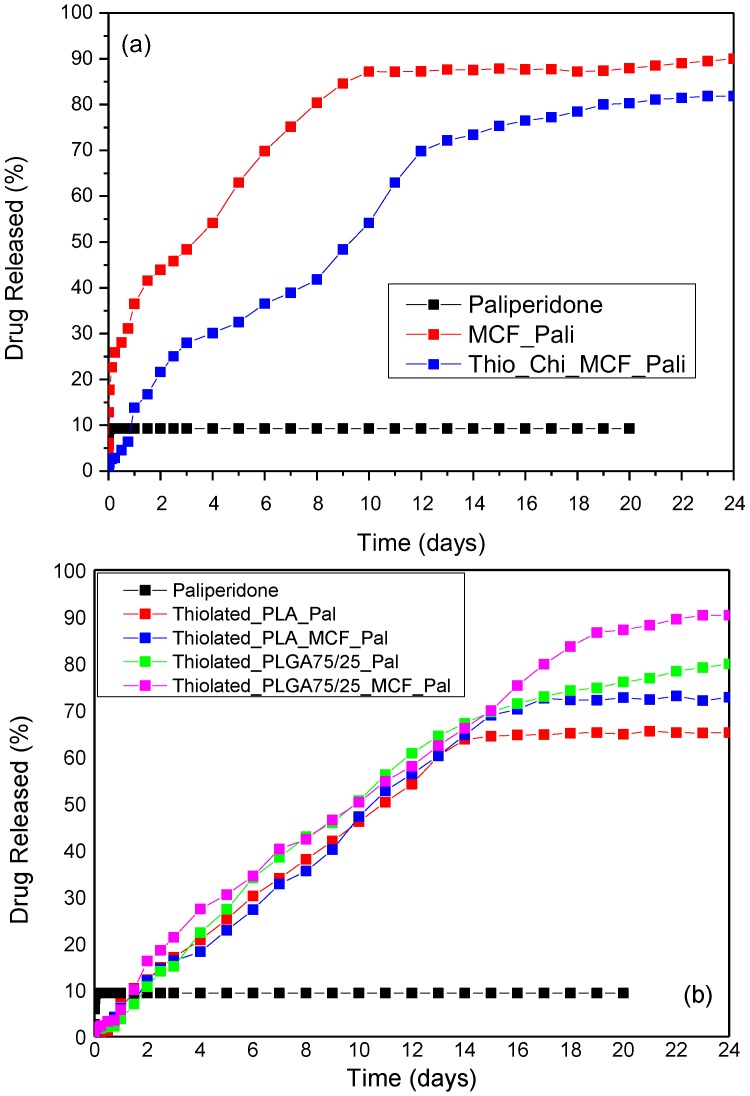
Dissolution profile of (**a**) Thiolated_ MCF_paliperidone and (**b**) Thiolated microspheres.

Figure 10 shows the dissolution profiles of MCF with adsorbed paliperidone and thiolated coated nanoparticles. As can be seen, the dissolution profile of neat paliperidone reached its maximum at about 10% in the first hour, without any further dissolution up to 20 days. This is clear proof that paliperidone is a poorly water soluble drug. When it is incorporated in MCF nanopores, the dissolution rate is substantially enhanced and it seems that after 10 days almost 90% of the encapsulated drug was released. This is due to the drug amorphization [44,45]. Thiolated MCF/paliperidone also showed an enhanced dissolution drug profile compared with neat paliperidone, and after 24 days about 80% of the drug was released. From these release studies it is clear that thiolation slightly reduces paliperidone release compared to MCF/paliperidone nanoparticles. This delay is also recorded from the beginning since in the first 6 h no significant drug released was observed; i.e., only 2.8%, in thiolated MCF/paliperidone nanoparticles. This is probably due to the entrance of thiolated chitosan into MCF pores during coating, which can close the gates of pores and prevent drug release from MCF. Also, the thiolated coating layer that formed on the microparticles’ surface (Figure 4c) can further delay the drug release.

Figure 10b shows the dissolution of paliperidone from thiolated microspheres using PLA and PLGA75/25 *w*/*w* polymers as matrices. It seems that the masking of microparticles with thiolated chitosan reduces paliperidone’s release, compared to uncoated ones [18], probably due to the addition of a coated layer that acts as a barrier. Dissolution profiles showed that in the first 6 h no significant drug release occurred, probably because at that period swelling of thiolated chitosan may take place, delaying the drug release. This also causes the disappearance of the burst effect that appears in loaded microspheres without the thiolated chitosan coating layer [18]. After that, there is a continuous and sustained release paliperidone from all microspheres up to 14–15 days. A decrease in the dissolution rate was observed after that, depending on the microsphere used. As can be seen, the release is lower in PLA microspheres than in PLGA. As was found in our previous study, the drug release rate from polymeric microspheres depends on the melting point and glass transition of the polymers used [18]. So, this lower rate of paliperidone release from PLA microspheres should be attributed to its high *T*_g_ (61–64 °C) compared with the lower *T*_g_ of PLGA (52–55 °C). Furthermore, in both type of microspheres those with MCF have higher release rates, compared with microspheres where only paliperidone was dispersed. This could be attributed to the higher available surface on which the drug is dispersed, since MCF have a very high specific surface area (445 m^2^/g).

### 3.6. Kinetic Analysis of Drug Release

An extensive discussion of the release mechanisms and description of corresponding kinetics for the systems examined here in the absence of the thiolated chitosan layer has been given in a previous work [18]. So the analysis in the following will be mainly focused on the influence of the coating layer. Starting with the case of bulk paliperidone release, it was found that it is a simple dissolution process dominated by paliperidone solubility in water. The fast release curve shown in Figure 10a was very accurately described by considering an appropriate combination of dissolution rate constant and mass transfer coefficient. Before we proceed to the discussion of release from other materials, let us discuss what is expected to be the effect of the thiolated chitosan layer.

There are some studies on swelling and diffusion in chitosan derivatives [47,48]. At first the water diffuses into the chitosan layer, inducing its swelling. The swelled chitosan is a gel containing pores with water allowing pore diffusion of solutes. So the transient diffusion equation for paliperidone in the swelled layer combined with the diffusion of water in the dry layer determines the additional transport of paliperidone. It is noticed that the coating layer is initially free of paliperidone, so its dynamic characteristics can be deduced from the delay in the release process. In the present experiments this delay is always less than 0.7 days, which is relatively small compared to the characteristic overall release time (more than 10 days). This difference between the time scales suggests that the dynamic phenomena in the coating layer can be ignored and the effect of coating can be simply accounted as an additional resistance to paliperidone diffusion through the particles.

**Figure 11:**
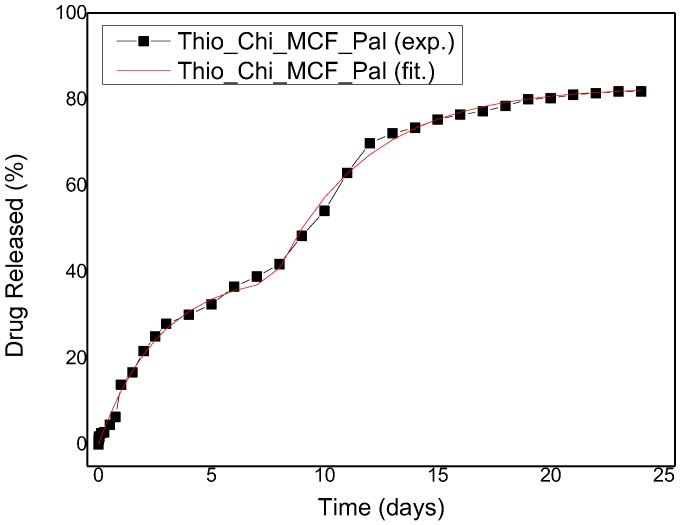
Paliperidone release profile for thiolated MCF particles. The experimental data are shown as symbols and the fitting data as a continuous line.

The next step is to analyze the release from coated MCF particles. Here, the process is desorption accompanied by diffusion, first through the MCF particle and then through the chitosan layer. In the previous work it was shown that the release curves from MCF particles cannot be described assuming structural homogeneity (i.e., a single apparent diffusion coefficient for the whole particle). Instead, three different diffusion coefficients corresponding to different fractions of the adsorbed paliperidone were considered. The first fraction is assumed to extend to the whole volume of the particles so the complete series solution of the transient diffusion equation is employed (necessary for the capture of the initial diffusion burst). The other two fractions are assumed to be initially located in the interior of the particles so the approximating linear driving force equation is used to account for their diffusion [49]. As is obvious from Figure 10a, the addition of a chitosan layer considerably delays the paliperidone release. The initial burst is completely eliminated since there is no initial paliperidone in the outer (chitosan) layer. This suggests that the fit can be done using solely the linear driving force formula (LDF) (instead of the complete diffusion equation). An acceptable fitted curve results from assuming two different diffusion constants (instead of three in the absence of a chitosan layer). This is quite reasonable since the additional diffusion resistance of the coating layer shadows the internal inhomogeneities. The equation used for fitting is

(4) $$\frac{C}{C_{\infty }}=\varphi_{1}(1-\exp(-K_{1}t))+\varphi_{2}(1-\exp(-K_{2}U(t-\tau )))$$

where U(*x*) is a function defined as U = 0 for *x* ≤ 0 and U = *x* for *x* > 0. The coefficient values are φ_1_ = 0.48, Κ_1_ = 0.37 days^−1^, φ_2_ = 0.52, Κ_2_ = 0.24 days^−1^, τ = 7 days. The time shifts in the absence of chitosan layer were 0.75 and 3.7 days [18]. The first one is eliminated and the second is shifted to seven days due to the mass transfer resistance imposed by the chitosan layer. The extension of the LDF to account for a coating layer leads to the relation $K={\left(\frac{R^{2}}{15D_{p}}+\frac{R\delta }{3D_{c}}\right)}^{-1}$, where *R* is the MCF particles’ radius (an average value is considered), δ is the chitosan layer thickness, and D_p_, D_c_ are the apparent diffusion coefficients in the particle and in the chitosan layer, respectively. The particle diffusivity should not depend on the existence of the coating layer so the values of D_p_ from fractions 2 and 3 found in the previous work can be employed in order to find the chitosan layer diffusivity D_c_. Assuming a layer thickness of 40 nm, the result is D_c_ = 1.84 × 10^−20^ m^2^/s. This value is on the same order as the diffusivities in the MCF particles [18], which means that the primary effect of the coating layer in this case is the creation of an initial solute-free zone outside the particles and the secondary effect is the additional mass transfer resistance. The comparison between the experimental and fitted release curves is shown in Figure 11.

**Figure 12:**
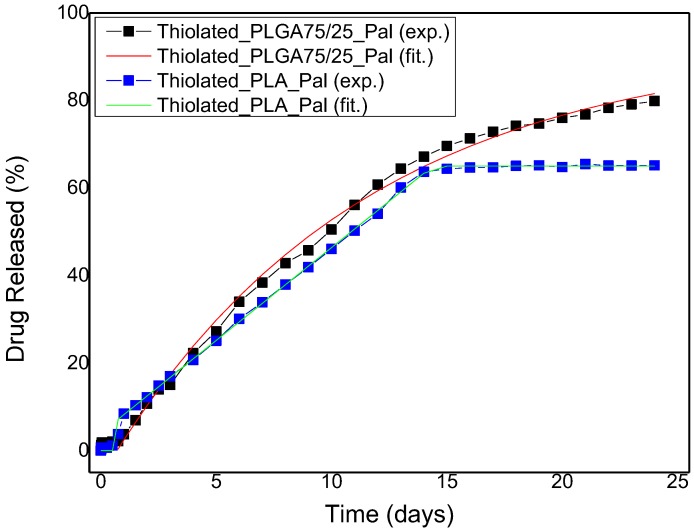
Paliperidone release profile for thiolated PLGA and PLA microspheres. The experimental data are shown as symbols and the fitting data as a continuous line.

The next step is the analysis of the release curves from the coated polymer microspheres. In our previous work we found that the release from the uncoated microspheres can be described as a Fickian diffusion process [50]. The complete solution of the transient diffusion equation was successfully used to fit the data (including the initial release burst). In the presence of chitosan coating for PLGA, the initial burst disappears and the whole release process is delayed (similar to the MCF chitosan-coated particles). The process can be described in this case using a simple LDF formula:

(5) $$\frac{C}{C_{\infty }}=1-\exp(-KU(t-\tau )),$$

where τ accounts for the initial dead time due to the existence of the (initially empty of solute) chitosan layer. The fit to the data gives τ = 0.7 days and K = 0.09 days^−1^. This value is compatible with the relation $K={\left(\frac{R^{2}}{15D_{p}}+\frac{R\delta }{3D_{c}}\right)}^{-1}$, where R is the microsphere radius (the typical value of 1.3 μm is used here), δ is the chitosan layer thickness of 40 nm, D_p_ is the diffusion coefficient of paliperidone in PLGA microspheres found in the previous work (D_p_ = 2.64 × 10^−18^ m^2^/s), and D_c_ is the diffusion coefficient of paliperidone in the coating layer found by analyzing the coated MCF particles’ release data. Whereas the coated PLGA data are compatible with the uncoated PLGA and the coated MCF release data, the situation is completely different for the coated PLA microspheres data. The release curve can be described as a simple line, which means that the release rate is constant up to the disappearance of the paliperidone from the particles. However, it has been shown [18] that the dominant mechanism for release from uncoated PLA particles is diffusion. In addition, in the present work it is shown that the coating layer acts as an additional diffusion resistance. The only explanation for having a constant rate process for the coated particles (instead of a diffusion one as in the PLGA case) is an interfacial transport barrier in the boundary between PLA and coating. This barrier exhibits a zero-order kinetic, i.e., the rate of transport from PLA to coating does not depend on the solute concentration in PLA. Such a barrier does not exist in the PLGA–coating interface. From a practical point of view the achievement of a constant release rate in the PLA-coating system is a major issue since release rate uniformity is almost impossible to achieve for a diffusion-dominated system [51]. The comparison between fitting and experimental coated polymer microspheres release curves is shown in Figure 12.

**Figure 13:**
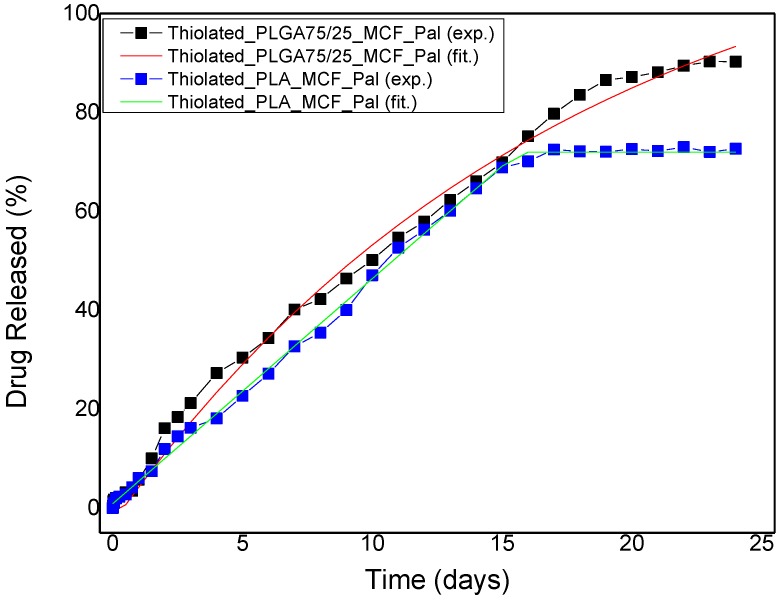
Paliperidone release profile for thiolated PLGA/MCF and PLA/MCF microspheres. The experimental data are shown as symbols and the fitting data as a continuous line.

Let us finally discuss the release curves for the combined MCF/polymer/coating system. In the absence of a coating, the composite particle release showed some resemblance to MCF particle release, which was physically and mathematically described by the presence of MCF particles on the surface of composite particles [18]. Here the coating layer excludes this possibility and this is evident in the release curves that have no similarity with the MCF particle release profile. The effect of the coating layer is dominant, such that the addition of MCF particles into the polymer particles has no actual influence on the shape of the controlled release curves. The fitting equations are the same as those used for the coated polymer particles and the fitting results are shown in Figure 13. As a confirmation of the dominance of the PLA–coating layer interface transport on the release rate, it is noted that the release rate is the same in the absence and presence of MCF particles in the polymer particle.

## 4. Conclusions

In this study thiolated chitosan was used to mask microspheres of PLA and PLGA 75/25 *w*/*w* containing MCF silica loaded with paliperidone drug. The main scope was to evaluate any possible changes that happened in microspheres after their masking with thiolated chitosan, a mucoadhesive polymer that is used for intranasal delivery formulations. In all cases polymeric microspheres with spherical shapes were formed, with sizes varying between 1 and 3 μm. In XRD studies it was found that paliperidone was encapsulated in an amorphous form inside of MCF pores. SEM and TEM images showed that thiolated chitosan formed a film that coated the microspheres. As was found from drug release studies and model fitting of release rates, this film coating resulted in the delay of the dissolution release profile of paliperidone. Also, it was found that, due to this coating layer, the addition of MCF particles into the polymer microspheres had no influence on the drug release rate. However, the encapsulation of paliperidone into MCF nanoparticles is essential for drug dissolution enhancement.

## Figures and Tables

**Table 1:** Microparticles yield (%), drug loading (%), and entrapment efficiency (%) of prepared thiolated coated microparticles.

Sample	Microparticle Yield (%)	Drug Loading (%)	Entrapment Efficiency (%)
Thiolated_PLA_Pal	85.95 ± 4.94	8.65 ± 1.90	36.25 ± 3.03
Thiolated_PLA_MCF_Pal	93.64 ± 6.17	18.09 ± 4.45	33.62 ± 2.43
Thiolated_PLGA75/25_Pal	90.43 ± 1.99	9.47 ± 2.79	47.77 ± 5.98
Thiolated_PLGA75/25_MCF_Pal	94.70 ± 3.12	21.92 ± 1.35	41.27 ± 5.06
